# Supplementary material for: Drug-related problems in hypertension and gestational diabetes mellitus: A hospital cohort
Source: PLoS One. 2023 Apr 7;18(4):e0284053. doi: 10.1371/journal.pone.0284053 (PMC10081748; doi:10.1371/journal.pone.0284053)
Supplement: S1 Table — (DOCX) [file pone.0284053.s001.docx]

Table S1 - Parameters Used for Classification of Effectiveness and Safety in Drug Related Problems (DRP)

| **ATC Class** | Criteria for DRPs adopted | |
| --- | --- | --- |
|  | **Effectiveness (P1)** | **Safety (P2)** |
| J01C BETA-LACTAM ANTIBACTERIALS, PENICILLINS | No clinical improvement within 48 hours of administration, axillary temperature > 37.6 °C and total leukocytes > 11,000 mm³ | Occurrence of IgE mediated allergic reactions within 4 hours post administration, dermatological and gastrointestinal reactions |
| N02B OTHER ANALGESICS AND ANTIPYRETICS | Absence of analgesia 2 hours after administration | Occurrence of gastrointestinal, dermatological, cardiovascular, hepatic (AST > 32 U/L OR ALT > 25 U/L OR GGT > 36 U/L) and endocrine/metabolic reactions |
| A04A ANTIEMETICS AND ANTINAUSEANTS | Maintained nausea 24 hours after administration or episodes of emesis | Complaint of blurred vision or sedation 30 minutes after re-administration |
| B01A ANTITHROMBOTIC | Lack of instructions for administering the drug at the proper time, which may imply lower absorption if administered together with food | Complaint of heartburn or headache 30 minutes post re-administration |
| B03B VITAMIN B12 AND FOLIC ACID | Lack of instructions for administering the drug at the proper time, which may imply lower absorption if administered together with food | Occurrence of hypersensitivity, dermatological and cardiovascular reactions |
| J01D OTHER BETA-LACTAM ANTIBACTERIALS | No clinical improvement within 48 hours post-administration, axillary temperature > 37.6 °C and total leukocytes > 11,000 mm³ | Complaint of diarrhea, heartburn, headache, gastralgia, emesis 30 minutes post re-administration |
| N06A ANTIDEPRESSANTS | No clinical improvement within 15 days of administration | Complaint of xerostomia, sedation, dizziness and nausea after 30 minutes re-administration |
| C02D ARTERIOLAR SMOOTH MUSCLE, AGENTS ACTING ON | Maintenance of systolic blood pressure ≥160 mmHg or diastolic blood pressure ≥110 mmHg within 24 hours post administration | Complaint of sedation and headache 30 minutes post re-administration |
| C03A THIAZIDE DIURETICS | Maintenance of systolic blood pressure ≥ 140 or diastolic blood pressure ≥ 90 mmHg in 48 hours post administration | Occurrence of serum potassium concentrations < 3.0 mEq/L (hypokalemia), dermatological toxicity, uric acid <6 mg/dL (hyperuricemia/gout and hypersensitivity reactions (immediate and delayed) |
| A02A [ANTACIDS](https://www.whocc.no/atc_ddd_index/?code=A02A&showdescription=no) | Maintenance of heartburn sensation within 24h post-administration | Occurrence of gastrointestinal, endocrine/metabolic reactions |
| A10A [INSULINS AND ANALOGUES](https://www.whocc.no/atc_ddd_index/?code=A10A&showdescription=no) | Suboptimal glycemic control/Drug dosing too low/Infrequent dosing regimen (Fasting ≥ 95 mg/dL and 1 hour postprandial ≥140 mg/dL or 2 hours postprandial ≥ 120 mg/dL or less) | Occurrence of glucose values < 70mg/dL (hypoglycemia) |
| C02A CENTRALLY ACTING ANTIADRENERGICS | Maintenance of systolic blood pressure ≥ 140 or diastolic blood pressure ≥ 90 mmHg in 48 hours post administration | Complaint of headache, sedation, dizziness or nausea 30 minutes post re-administration |
| C08C [SELECTIVE CALCIUM CHANNEL BLOCKERS WITH MAINLY VASCULAR EFFECTS](https://www.whocc.no/atc_ddd_index/?code=C08C&showdescription=no) | Maintenance of systolic blood pressure ≥ 140 or diastolic blood pressure ≥ 90 mmHg in 48 hours post administration | Complaint of headache, dizziness or nausea 30 minutes post re-administration |
| D01A ANTIFUNGALS FOR TOPICAL USE | Maintenance of itching and vaginal discharge 48 hours post administration | Occurrence of dermatological reactions and hypersensitivity |
| A06A [DRUGS FOR CONSTIPATION](https://www.whocc.no/atc_ddd_index/?code=A06A&showdescription=no) | Maintenance of constipation 24 hours post- administration | Occurrence of gastrointestinal reactions |
| C07A [BETA BLOCKING AGENTS](https://www.whocc.no/atc_ddd_index/?code=C07A&showdescription=no) | Maintenance of systolic blood pressure ≥ 140 or diastolic blood pressure ≥ 90 mmHg in 48 hours post administration | Occurrence of cardiovascular and nervous system reactions |
| A02B [DRUGS FOR PEPTIC ULCER AND GASTRO-OESOPHAGEAL REFLUX DISEASE (GORD)](https://www.whocc.no/atc_ddd_index/?code=A02B&showdescription=no) | Drug dose too high/ infrequent dosing regimen or no clinical improvement - 75 mg to 150 mg up to twice daily (maximum: 2 doses/day); do not use for longer than 14 days | Headache complaint 30 minutes after re-administration |
| A03A DRUGS FOR FUNCTIONAL GASTROINTESTINAL DISORDERS | Flatulence maintenance 24 hours post administration | Adverse reactions are less likely to occur because it is not absorbed by the body. It acts only inside the digestive tract, being totally eliminated in the feces, without alterations |
| B03A [IRON PREPARATIONS](https://www.whocc.no/atc_ddd_index/?code=B03A&showdescription=no) | Lack of instructions for administering the drug at the proper time, which may imply lower absorption if administered together with food | Complaint of dizziness or nausea 30 minutes post re-administration |
| N02A [OPIOIDS](https://www.whocc.no/atc_ddd_index/?code=N02A&showdescription=no) | Maintenance of pain within 24 hours post administration | Complaint of blurred vision or constipation 2 hours post re-administration |
| H02A [CORTICOSTEROIDS FOR SYSTEMIC USE, PLAIN](https://www.whocc.no/atc_ddd_index/?code=H02A&showdescription=no) | Pregnant woman out of risk of premature delivery within 7 days | Occurrence of hyperglycemia 30 min post administration (fasting blood glucose ≥ 95 mg/dL and 1 hour postprandial ≥140 mg/dL or 2 hours postprandial ≥ 120 mg/dL) |
| N05A ANTIPSYCHOTICS | Clinical improvement within 1 to 2 weeks of treatment | Complaint of sedation, dizziness or nausea 30 minutes post re-administration |
