# Supplementary material for: Drug-related problems in hypertension and gestational diabetes mellitus: A hospital cohort
Source: PLoS One. 2023 Apr 7;18(4):e0284053. doi: 10.1371/journal.pone.0284053 (PMC10081748; doi:10.1371/journal.pone.0284053)
Supplement: Attachment — Submitted filename: Response to reviewers.docx [file pone.0284053.s002.docx]

23th February 2023

Dear Dra Irles,

In this letter, we detail the changes introduced in the manuscript “***Drug-related problems in hypertension and gestational diabetes mellitus: a hospital cohort***” in response to the reviewers’ comments. All reviewers’ comments were accepted and we appreciate the important collaborations.

Yours sincerely,

Rand Randall Martins, PharmD. PhD.

Corresponding Author

***Journal Requirements***

1. Please ensure that your manuscript meets PLOS ONE's style requirements, including those for file naming. The PLOS ONE style templates can be found at

https://journals.plos.org/plosone/s/file?id=wjVg/PLOSOne_formatting_sample_main_body.pdf and <https://journals.plos.org/plosone/s/file?id=ba62/PLOSOne_formatting_sample_title_authors_affiliations.pdf>

*Response: The style template was readequate as suggested.*

2. Thank you for stating the following financial disclosure: “This study was financed in part by the Coordenação de Aperfeiçoamento de Pessoal de Nível Superior – Brasil (CAPES) - Finance Code 001”. Please state what role the funders took in the study. If the funders had no role, please state: ""The funders had no role in study design, data collection and analysis, decision to publish, or preparation of the manuscript.""

If this statement is not correct you must amend it as needed.

Please include this amended Role of Funder statement in your cover letter; we will change the online submission form on your behalf.

*Response:* *The cover letter was readjusted as suggested.*

3. Thank you for stating the following in the Acknowledgments/ Funding Section of your manuscript: “This study was financed by the Coordenação de Aperfeiçoamento de Pessoal de Nível 25 Superior – Brasil (CAPES) - Finance Code 001.”

We note that you have provided funding information that is not currently declared in your Funding Statement. However, funding information should not appear in the Acknowledgments section or other areas of your manuscript. We will only publish funding information present in the Funding Statement section of the online submission form.

Please remove any funding-related text from the manuscript and let us know how you would like to update your Funding Statement. Currently, your Funding Statement reads as follows:

“This study was financed in part by the Coordenação de Aperfeiçoamento de Pessoal de Nível Superior – Brasil (CAPES) - Finance Code 001”

Please include your amended statements within your cover letter; we will change the online submission form on your behalf.

*Response: The manuscript has been corrected as per the suggestions.*

4. Please review your reference list to ensure that it is complete and correct. If you have cited papers that have been retracted, please include the rationale for doing so in the manuscript text, or remove these references and replace them with relevant current references. Any changes to the reference list should be mentioned in the rebuttal letter that accompanies your revised manuscript. If you need to cite a retracted article, indicate the article’s retracted status in the References list and also include a citation and full reference for the retraction notice.

*Response: The references were thoroughly reviewed and corrected to align with the formatting guidelines of the journal. In order to meet the publication criteria, minor changes were made to the formatting, including replacing one reference with a more current and relevant source. (page 25, line 478-486).*

*Deleted reference:*

~~Ghosh KR, Akhter S, Das AK, Naher N, Paul SR, Islam B. Outcome of Labetalol and Methyldopa as Oral Antihypertensive Agent in the Treatment of Pregnancy Induced Hypertension. Mediscope. 2021;8(1):19-26. doi: https://doi.org/10.339/mediscope.v8i1.52200~~

*Added reference:*

Van de Vusse D, Mian P, Schoenmakers S, Flint RB, Visser W, Allegaert K, et al. Pharmacokinetics of the most commonly used antihypertensive drugs throughout pregnancy methyldopa, labetalol, and nifedipine: a systematic review. Eur J Clin Pharmacol. 2022;78(11):1763-1776. doi: 10.1007/s00228-022-03382-3

5. Statistical analysis, sentence: “The sample size was set at 600 individuals, which ensures a maximum error of the estimates of ± 4 percentage points with 95% confidence.” Was the sample size determined a priori or post hoc? Provide details about how 4% was calculated (including the software/formula used).

*Response: The text was changed to (page 8, line 163-169):*

*“The sample size was set at 600 individuals, which ensures a maximum error of the estimates of ± 4 percentage points with 95% confidence. We employed the following formula to calculate the sample size:*

$$Sample size=\frac{{Z_{1-\propto/2}}^{2}p(1-p)}{d^{2}}$$

*Here:*

*Z _1- α/2_ = 1.96 (standard normal variable considering a type 1 error of 5% and p <0.05).*

*p = Expected proportion of DRP in the population. As there are no similar studies available, we opted for a proportion that enables the largest sample size (50%).*

*d = Absolute error (4%).”*

6. In statistical analysis, the authors mentioned absolute and relative frequency. Provide additional explanation. Did the authors report relative frequency in the manuscript?

*Response: Thanks for the observation, the term used was imprecise. The text was changed to (page 8, line 173):*

*“The descriptive statistics included the median and 25th and 75th percentiles (p25-75%); ~~relative and~~ absolute frequency and percent proportion; and mean and standard deviation according to the type of variable under analysis.”*

7. In statistical analysis, state the method used for selecting the variables that were included in the multivariable regression analysis.

*Response: The text was changed to (page 9, line 182-188):*

*“~~All variables with an association test with p-value <0.10 were included in a multivariate logistic regression model, and a significance level of p<0.05 was then adopted to identify the factors independently associated with the occurrence of DRPs in pregnant women.~~ Variables that exhibited a significant association with a p-value < 0.10 were included in a multivariate logistic regression model. The stepwise backward variable selection method, with a significance level of p < 0.05, was used to identify independent factors associated with the occurrence of DRPs in pregnant women.”*

***Reviewer #1***

8. Thank you for asking me to review this article. The article is not only interesting but quite informative as it appears to bring to our consciousness some of the obscure factors affecting drug effectiveness in high risk pregnancy. The methodology is appropriate for the research objectives. The use of a single institution for data collection seems to be a major concern, but this can be addressed with a multi centre study in future. I would recommend this article for publication.

***Reviewer #2***

9. In the abstract, the following, the univariate analysis is not mentioned. Also Insulin and methyldopa are not pharmacological classes. Please check and correct.

*Response: Following the reviewer's suggestion, the text was changed to (page 3, line 51-56):*

*“In addition to descriptive statistics, a univariate and multivariate logistic regression model was employed to determine the factors associated with the DRPs. Results: A total of 873 DRPs were identified. The most frequent DRPs were related to therapeutic ineffectiveness (72.2%) and occurrence of adverse events (27.0%) and the main drugs and the main pharmacological classes involved were insulins and methyldopa.”*
